# Bcl-2 protein family expression pattern determines synergistic pro-apoptotic effects of BH3 mimetics with hemisynthetic cardiac glycoside UNBS1450 in acute myeloid leukemia

**DOI:** 10.1038/leu.2016.341

**Published:** 2017-01-03

**Authors:** C Cerella, A Gaigneaux, A Mazumder, J-Y Lee, E Saland, F Radogna, T Farge, F Vergez, C Récher, J-E Sarry, K-W Kim, H Y Shin, M Dicato, M Diederich

**Affiliations:** 1 Laboratoire de Biologie Moléculaire et Cellulaire du Cancer, Hôpital Kirchberg, Luxembourg City, Luxembourg; 2 Department of Pharmacy, College of Pharmacy, Seoul National University, Seoul, Korea; 3 Cancer Research Center of Toulouse, UMR 1037 INSERM/Université Toulouse III-Paul Sabatier, Oncopole, Toulouse, France; 4 Université Toulouse III-Paul Sabatier, Toulouse, France; 5 Service d'Hématologie, Centre Hospitalier Universitaire de Toulouse, Institut Universitaire du Cancer de Toulouse Oncopole, Toulouse, France; 6 SNU-Harvard Neurovascular Protection Center, College of Pharmacy and Research Institute of Pharmaceutical Sciences, Seoul National University, Seoul, Korea; 7 Department of Pediatrics, Cancer Research Institute, Seoul National University College of Medicine, Seoul, Korea

Resistance to apoptotic cell death^1^ owing to overexpression of anti-apoptotic Bcl-2 family proteins including Bcl-2, Bcl-xL or Mcl-1 is considered an interesting druggable target for the treatment of hematological malignancies including acute myeloid leukemia. In fact, BH3 mimetics^2^ like ABT-199 (venetoclax)^3^ reverse the inhibitory function of anti-apoptotic Bcl-2 proteins.^4^ Dependency on Bcl-2 family protein expression requests BH3 profiling to efficiently stratify patients potentially benefiting from ABT-199 therapy.^5^ Most often, Mcl-1 is considered a main resistance factor^2^ and recently a first class of selective Mcl-1 inhibitors was characterized.^6^ As an alternative to functional inhibitors, we previously described proteasome-dependent downregulation of Mcl-1 expression^7, 8^ by cardiac glycoside UNBS1450.^8, 9, 10^ We hypothesize here that a combination of UNBS1450 with a BH3 mimetic would affect acute myeloid leukemia (AML) subtypes especially ‘addicted' to Mcl-1. To provide a targeted therapeutic approach, we describe the synergistic antileukemic effect of ABT-199 with UNBS1450 in cell lines, colony formation assays, zebrafish xenografts and validate results in primary cells from 23 *de novo* AML patients.

Figure 1a shows the expression pattern of major anti-apoptotic Bcl-2 proteins of selected AML cell lines suitable for the assessment of single/combinational strategies. First, we assessed the sensitivity of these cells against ABT-199, ABT-263 and UNBS1450 as a single agent, by determining inhibitory concentration (IC_50_) values after 24 h (Figure 1b). A multiple linear regression for each drug on the three proteins (Figure 1c) confirmed the significant positive correlation between IC_50_ values and Bcl-2 expression and, *vice versa*, a negative correlation with Mcl-1 expression for ABT-199. IC_50_ values positively correlated with Mcl-1 expression and negatively with Bcl-xL expression for UNBS1450, in agreement with previous data.^8^ For ABT-263, we could not reach significance based on this panel; analysis of the raw data indicates a positive correlation with Bcl-2 and Bcl-xL expression, and a negative one with concomitant Mcl-1 expression. Generally, effects with ABTs were obtained with micromolar concentrations, prone to trigger resistance.

We then selected U937 and TF-1 cell lines as models to investigate the synergistic potential of combination treatments. Mcl-1 inhibitor A-1210477 previously allowed characterizing Bcl-2 protein co-dependency involving Mcl-1.^11^ Combination of A-1210477 and ABTs provided evidence of the co-requirement of Bcl-2/Mcl-1 expression and Bcl-xL/Mcl-1 in U937 and TF-1 cells, respectively (Supplementary Figure 1). A-1210477 primed TF-1 cells (highly co-expressing Bcl-xL/Mcl-1) to apoptosis only when combined with ABT-263. We then replaced A-1210477 by UNBS1450. In U937 cells, we documented a strong synergistic effect when UNBS1450 was combined with both ABTs (confidence interval=0.14–0.18; Figure 1d; Supplementary Figure 2). In TF-1 cells, synergism was observed only when combining UNBS1450 with ABT-263, targeting Bcl-2, Bcl-xL and Bcl-w, but not with ABT-199, selective for Bcl-2, undetectable in these cells (confidence interval=0.05–0.15; Figure 1e).

Colony formation was strongly reduced when U937 cells were treated with a combination of UNBS1450/ABT-199 (Supplementary Figure 3), whereas tumor mass formation was completely abrogated in a zebrafish xenograft model, whereas individual treatments did not, validating our results (Figure 2a).

We confirmed differential toxicity by a combined treatment (20 nm UNBS1450; 0.1 μm ABT-199) that led to 40% induction of cell death (Figure 2b; Supplementary Figure 3A), but of 80% with ABT-263 (Supplementary Figure 4B) in CD34^+^ cells from cord blood of healthy donors, compared with 100% in leukemia cells.

As platelets were strongly affected by ABT-263,^2, 3^ we tested different concentrations of UNBS1450 alone or in combination with ABT-199 without impacting the viability of leukocyte-depleted platelets pool from healthy donors.^3, 12^ ABT-263, used as reference, deteriorated viability (Figure 2c and Supplementary Figure 4C).

Next, we analyzed UNBS1450 alone and combined to ABT-199 on 23 *de novo* diagnosed AML patients (Figure 2, Supplementary Figure 5, Supplementary Table 1). UNBS1450 dose- and time-dependently reduced viability of primary AML cells (Figure 2d). A subgroup of 14 AML patients moderately responded to UNBS1450 or ABT-199 alone, but were sensitized to death in co-treatments (Figure 2e). Analysis of CD34^+^CD38^−^ subpopulations confirmed these results (Figures 2d and e). In the same AML samples, no significant impact on healthy lymphocytes was observed (Figure 2f). Analysis of expression patterns of major anti-apoptotic Bcl-2 family members in the panel of AML patients revealed the presence of a second band for Mcl-1 in many specimens, which is compatible with the reported 32 kDa short isoform. This band was not or barely detectable in established AML cell lines (Figure 1a; Supplementary Figure 7). A multiple correspondence analysis (Supplementary Figure 8) based on the expression level of Bcl-2, Bcl-xL and both detectable Mcl-1 bands (40 and 32 kDa) showed that patient samples that are sensitive to ABT-199 (Pt no. 1, 6, 9 and 18) exhibit a high expression level of Bcl-2, as expected, but also of Mcl-1 32 kDa, besides Bcl-xL. A group of AML patient cells highly susceptible to UNBS1450 (Pt no. 4, 5, 8, 11 and 19) generally present a reduced level of Bcl-xL and high expression levels of Mcl-1 32 kDa. We could not associate any typical profile with samples positively responding to the co-treatment; however, most samples belonging to this group concomitantly express Mcl-1 and Bcl-2 proteins at various levels (Pt no. 3, 12, 14, 16 and 17; Figure 2g).

Our results can provide the basis for future clinical trials with UNBS1450 used as single agent or in combination with ABTs in AML. Bcl-2 protein expression patterns, especially Mcl-1, could become an essential biomarker allowing AML patient stratification and response prediction. Results also prompt to explore both role and/or origin of the different Mcl-1 isoforms in drug response, a novel and emerging topic. A few studies focus on the modulatory roles of short isoforms and the actual biological functions of the 32 KDa Mcl-1 short isoform were recently investigated.^13, 14^ Moreover, from a mechanistic point of view, UNBS1450-induced degradation of Mcl-1 occurs via proteasome-dependent, noxa-independent degradation of preexisting Mcl-1 rather than by a transcriptional modulation of Mcl-1 expression.^8^

Further studies are required to validate subtype dependency on Bcl-2 protein expression more likely to benefit from a combination treatment. Recent application of BH3 profiling on patient samples allows establishing Bcl-2 dependency and predicting ABT response profile.^15^ The same approach can be applied to predict patients responsive to combination treatments.

So far, our analysis was carried out on *de novo* AML. We will extend our investigations to relapsed forms. Moreover, the patient panel tested so far does not allow correlations with specific factors including age, sex or cytogenetic mutations. An exploratory study to correlate specific mutations to drug response indicates that FLT3-ITD mutation has not the same effect for all treatments as opposed to FLT3-WT (Supplementary Figure 6): it decreases susceptibility to ABT-199 (−16%) and combination treatments (−12%), whereas increasing susceptibility to UNBS1450 (+20%). Even though none of these effects is significant in our limited panel, however, this preliminary evidence encourages further studies on effects of UNBS1450 alone or in combination with ABTs on FLT3-ITD AML patients' subgroups.

Importantly, we observed here that subtoxic single treatments by ABT compounds do not change anti-apoptotic Bcl-2 protein expression. Moreover, UNBS1450 downregulates Mcl-1 without a compensatory overexpression of other Bcl-2 family proteins (Figure 1d).

## Figures and Tables

**Figure 1:**
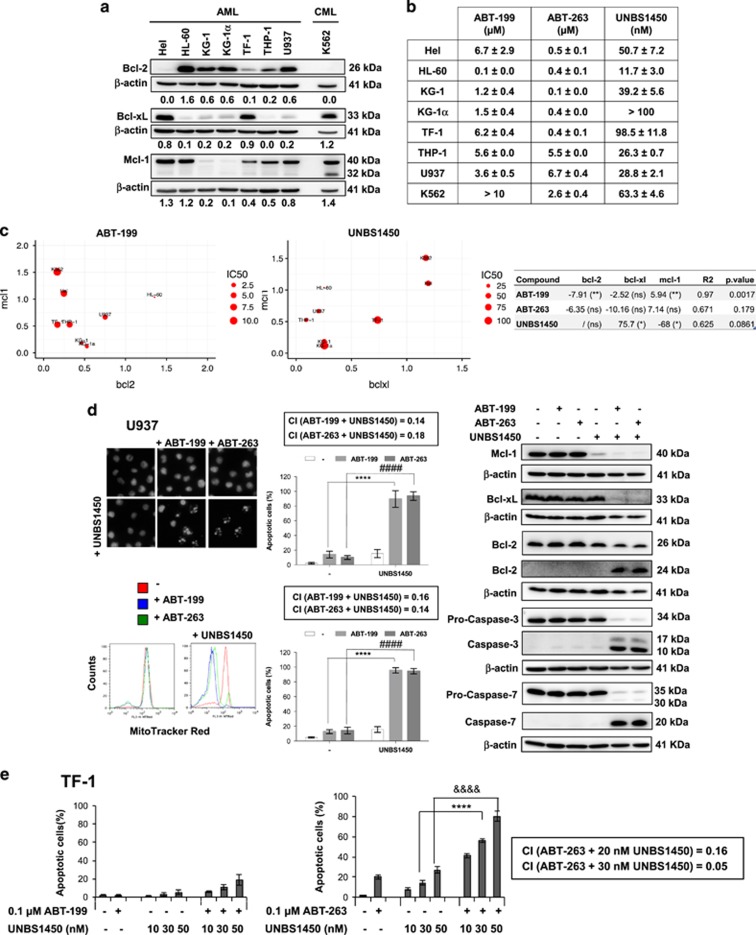
Correlation between cell-type specific expression of anti-apoptotic Bcl-2 family proteins and synergistic combinational effects of ABTs and UNBS1450 in AML cells. (**a**) Analysis of major anti-apoptotic Bcl-2 proteins in AML cell lines. Chronic myeloid leukemia (CML) K562 cells were used for comparative studies. (**b**) Susceptibility to ABTs and UNBS1450 after 24 h incubation. IC_50_ values determined by mitochondrial membrane potential loss and quantification of apoptotic nuclear morphology. (**c**) Effect of significant protein expression levels (β-actin ratios) on IC_50_ for ABT-199 (μm) and UNBS1450 (nm). The larger the size, the larger IC_50_. Coefficients estimated from multiple regression equations (right), *P<0.05; **P<0.01. (**d**) Synergistic effects of subtoxic concentrations of ABTs (0.1 μm) and UNBS1450 (20 nm) in U937 cells assessed as described, together with western blot analysis of caspase cleavage in parallel to modulation of anti-apoptotic Bcl-2 proteins. (**e**) TF-1 cells treated at indicated concentrations of UNBS1450. Combinational index (CI) was estimated by Calcusyn software (Biosoft, Cambridge, UK). Data are the mean of at least three independent experiments±s.d. Significance was estimated by using two-way anaylsis of variance test (*post hoc* analyses, Dunnett). Significance is reported as ****P<0.0001, ^####^*P*<0.0001.

**Figure 2:**
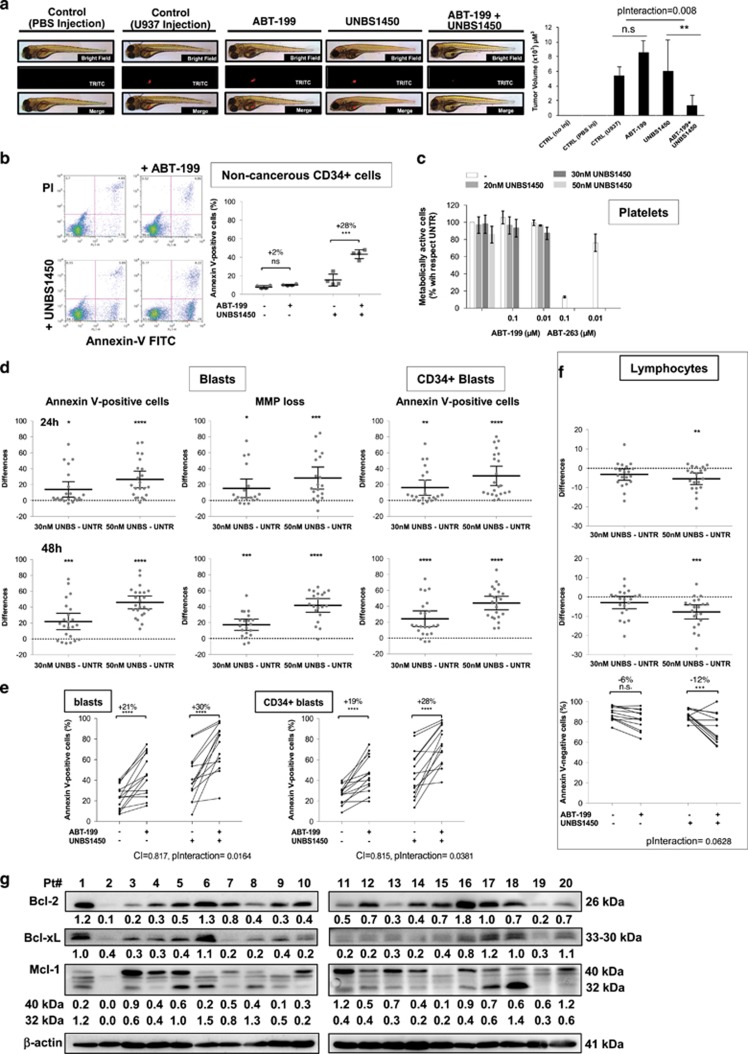
Impact of UNBS1450 alone or with ABTs. (**a**) Effect of UNBS1450 and ABT-199 single/combination treatments on tumor formation in a zebrafish xenograft model with U937 cells injected after an 8 h UNBS1450/ABT-199 pretreatment. Analysis of differential toxicity in healthy donor CD34^+^ (**b**) and platelets (**c**) by Annexin-V (BD Pharmingen, Erembodegem, Belgium) and Cell Titer Glo assays (Promega, Leiden, The Netherlands), respectively. Apoptogenic potential of UNBS1450 alone or in combination on AML patient samples (Supplementary Data): (**d**) analysis of UNBS1450 alone (by Annexin-V assay or MitoTracker Red staining (Invitrogen, Thermo Fisher Scientific, Asse, Belgium)); (**e**) combination of UNBS1450 (30 nm; 48 h preincubation) and ABT-199 (0.01 μm; 18 h of incubation). (**f**) Same analysis in AML patient lymphocytic subpopulation. (**g**) Western blot analysis of anti-apoptotic Bcl-2 protein expression. Synergy was estimated by using the ‘response additivity' approach. Corresponding combinational index (CI) of significant interactions were computed. Statistical analyses were performed in GraphPad Prism (GraphPad Software Inc., La Jolla, CA, USA). Significance is *P<0.05; **P<0.01; ***P<0.001; ****P<0.0001 (two-way analysis of variance; repeated measures; *post hoc* analyses Dunnett; Sidak).
