# Supplementary material for: Bcl-2 protein family expression pattern determines synergistic pro-apoptotic effects of BH3 mimetics with hemisynthetic cardiac glycoside UNBS1450 in acute myeloid leukemia
Source: Leukemia. 2017 Jan 3;31(3):755–9. doi: 10.1038/leu.2016.341 (PMC5339427; doi:10.1038/leu.2016.341)
Supplement: Supplementary Table 1 [file leu2016341x1.docx]

**Suppl. Table 1.** Clinical characteristics of AML patients tested in this study.

(FAB, French-American-British, FLT3-ITD, FMS-like tyrosine kinase-3-internal tandem duplication; CEBPA, CCAAT/enhancer-binding protein alpha; NPMc, cytoplasmic nucleophosmin 1)

| **#** | **Sex/**  **age** | **FAB** | **Cytogenetic abnormalities** | **FLT3-ITD mutation** | **CEBPA** | **NPMc** |
| --- | --- | --- | --- | --- | --- | --- |
| 1 | F/51 | 1 | - | 0 | 0 | 1 |
| 2 | M/62 | 2 | - | 0 | 0 | 0 |
| 3 | F/21 | 5 | - | 1 | 0 | 1 |
| 4 | F/65 | 4 | - | 1 | nd | 0 |
| 5 | M/47 | 5 | - | 1 | 0 | 1 |
| 6 | M/54 | 2 | - | 0 | nd | 1 |
| 7 | M/74 | 1 | 47,X,-Y,t(2;8)(p22;q24),+4,+8x3,add(9)(q21),-11,-17,2~21 dmin[10] / 46,X,-Y,add(3)(q28),+8,+add(8)(q24),-11,-17,+der(?)hsr(?)[cp3] | 0 |  | 0 |
| 8 | F/35 | 4 | - | 1 | 0 | 0 |
| 9 | M/63 | 2 | - | 0 | 1 | 0 |
| 10 | F/63 | 5 | 46, XX, t(11;12)(p15;p13) | 0 | 0 | 0 |
| 11 | F/72 | 5 | 47, XX, +8 | 1 |  | 0 |
| 12 | M/53 | 4 | 46, XY, inv (16)(p13q22) | 0 |  | 0 |
| 13 | M/71 | 5 | - | 1 |  | 1 |
| 14 | M/52 | 4 | 47,XY,inv(16)(p13q22),+22<15>/46,XY<1> | nd | nd | nd |
| 15 | M/73 | 1 | - | 0 | nd | 0 |
| 16 | M/77 | 2 | - | nd | nd |  |
| 17 | F/25 | 5 | + | 0 |  | 0 |
| 18 | M/68 | 1 | - | 1 |  | 0 |
| 19 | M/53 | 5 | 46,XY,t(11;17)(q23;q12)<19>/46,XY<1> | nd | nd | nd |
| 20 | F/64 | 5 | 46,XX,[20] | 0 | 0 | 1 |
| 21 | M/72 | 4 | - | 0 |  | 0 |
| 22 | M/63 | 1 | 43,XY,add(4)(q31),-5,del(7)(q21q35) or add(7)(q21),-11,t(14;19)(q13;q13),-16,add(17)(p11),-20,+mar[cp5]/45,sl,-add(4),+4,add(8)(p12),+add(8)(p12),+11[cp6]/46,XY[1] | 0 |  | 0 |
| 23 | M/60 | 2 | - | 0 | 0 | 0 |
