# Supplementary material for: Bcl-2 protein family expression pattern determines synergistic pro-apoptotic effects of BH3 mimetics with hemisynthetic cardiac glycoside UNBS1450 in acute myeloid leukemia
Source: Leukemia. 2017 Jan 3;31(3):755–9. doi: 10.1038/leu.2016.341 (PMC5339427; doi:10.1038/leu.2016.341)
Supplement: Supplementary Information [file leu2016341x2.docx]

**Materials and Methods**

**Cell lines and treatments**

Acute myeloid leukemia (AML) cell lines Hel, HL-60, KG-1 KG-1α, TF-1, THP-1 and U937 were treated for 24 hours at different concentrations of ABT-199 or ABT-263 (0.1-10 µM) and UNBS1450 (5-100 nM) before assessing the effects on cell death. IC_50_ values were determined with two different approaches, quantification of apoptotic cells by nuclear morphology upon Hoechst staining (Sigma-Aldrich, Bornem, Belgium) and mitochondrial membrane potential loss assessed upon MitoTracker® Red staining (Molecular Probes, Invitrogen, Fisher Scientific, Tournai, Belgium) as previously described.[^1^](#_ENREF_1)^,^ [^2^](#_ENREF_2)

All cell lines were purchased from DMSZ (Braunschweig, Germany). U937 and TF-1 cells were simultaneously treated for 18 hours with indicated concentrations of compounds before cell death analysis.

**Colony formation assay**

For colony formation assays, cells (10^3^ cells/ml) were treated for 10 hours with 30 nM UNBS1450 or 0.01 μM ABT-199, alone or in combination, and then grown in semi-solid methylcellulose medium (Methocult H4230, StemCell Technologies Inc., Vancouver, Canada). Colonies were detected after 10 days of culture by adding 1 mg/ml of 3-(4,5-dimethylthiazol-2-yl)-2,5-diphenyltetrazoliumbromide (MTT) reagent (Sigma) and were scored by Image J software (U.S. National Institute of Health, Bethesda, MD, USA).[^3^](#_ENREF_3)

**Zebrafish assay**

Wild type zebrafish (*Danio rerio*) were obtained from the Zebrafish International Resource Center (ZIRC, University of Oregon, OR), maintained according SNU guidelines at 28.5°C with 10hr dark/14hr light cycles. Viability and abnormal development were assessed under light microscopy (Carl Zeiss Stereo microscope DV4, Seoul, Korea). Pictures were taken by fixing zebrafish embryos onto a glass slide with 3% methyl-cellulose (Sigma Aldrich). For cancer xenograft assays, after mating, fertilized eggs were incubated in Danieau’s solution with 0.003% of phenylthiourea (PTU) at 28.5°C for 48hr. Micropipettes for injection and anesthesia were generated from a 1.0 mm glass capillary (World Precision Instruments, FL, USA) by using a micropipette puller (Shutter Instrument, USA). 48 hours post fertilization (hpf), zebrafish were anesthetized in 0.02% tricaine (Sigma, MO) and immobilized on an agar plate. 100-200 of U937 cells were stained for 2 hours by 4 μM of Cell tracker CM-Dil dye (Invitrogen), then treated with 30 nM UNBS1450 or 0.01 μM ABT-199, alone or in combination for 8 hours before injection into the yolk sac (PV820 microinjector, World Precision Instruments, FL, USA). Subsequently, zebrafish were incubated in 96-well plates containing Danieau’s solution with 0.003% phenylthiourea (PTU) at 28.5°C for 72 hr. Fish were then immobilized in a drop of 3% methylcellulose in Danieau’s solution on a glass slide. Pictures were taken by fluorescence microscopy (Leica DE/DM 5000B). Area of fluorescent tumors was quantified by Image J software (<http://rsb.info.nih.gov/ij/docs/index.html>).[^3^](#_ENREF_3)

**CD34^+^ isolation**

Healthy CD34+ cells were collected from human umbilical cord blood, kindly donated by Clinique Bohler (Luxembourg, Luxembourg), after written informed consent in agreement with the National Committee of Research Ethics in Luxembourg. Isolation of CD34+ cells was performed by using Ficoll^TM^ followed by magnetic cell sorting according to manufacturer’s instruction (Miltenyi, Utrecht, The Netherlands).[^4^](#_ENREF_4) Purity was around 96% as estimated by immunophenotyping and FACS analysis. After isolation, CD34+ cells were seeded at a concentration of 300,000/ml in serum-free medium (Stem Cell II, Sigma–Aldrich, Bornem, Belgium) supplemented with 10 ng/mL Interleukin 3 (IL3, Reliatech, Wolfenbuttel, Germany) and 10 ng/mL stem cell factor (SCF, Reliatech) for 3 days before performing experiments. Then cells were counted and diluted in fresh complete medium at a concentration of 300,000/ml before treatments (20nM UNBS1450 and 0.01μM ABT-199, alone and in combination for 18 hours in simultaneous treatment).

**Platelets**

Leucocyte-depleted platelet pools from 5 donors were kindly provided by Red Cross

Luxembourg (Luxembourg). Platelets were 1:10 diluted in RPMI medium and simultaneously treated at indicated compound concentrations for 24 hours.[^5^](#_ENREF_5) Viability of platelets was estimated by ATP quantification (Cell Titer-Glo Luminescent Assay, Promega, Leiden, The Netherlands) according to the manufacturer’s instruction.[^5^](#_ENREF_5) Data were recorded using an Orion Microplate Luminometer (Berthold Pforzheim, Germany), normalized to untreated cells and reported as a percentage of metabolically active cells. In parallel, percentage of Annexin-V cells was evaluated by using FITC Annexin V Apoptosis detection kit (BD Pharmingen, (Erembodegem, Belgium) [^6^](#_ENREF_6).

**AML primary samples**

Stored frozen samples from AML patients were obtained from the HIMIP collection (BB-0033-00060). According to the French law, HIMIP collection has been declared to the Ministry of Higher Education and Research (DC 2008-307 collection 1) and obtained a transfer agreement (AC 2008-129) after approbation by the “Comité de Protection des Personnes Sud-Ouest et Outremer II” (ethical committee). Clinical and biological annotations of the samples have been declared to the CNIL (Comité National Informatique et Libertés (Data processing and Liberties National Committee); samples were obtained from patients diagnosed with AML at the Toulouse University Hospital (TUH) after signed informed consent in accordance with the Declaration of Helsinki. Peripheral blood or bone marrow samples were frozen in fetal calf serum with 10% DMSO and stored in liquid nitrogen.

Cells were pre-incubated for 24 hours or 48 hours with the indicated concentrations of UNBS1450 before adding ABT-199 (0.01 µM) for further 18 hours and assessing cell death by Annexin-V assay or MitoTracker® Red staining at the FACS.

**Statistical analyses**

Statistical analyses were performed using Prism 6 software, GraphPad Software (La Jolla California, USA). Synergy was estimated by Calcusyn software [^7^](#_ENREF_7) whenever possible or by using the “response additivity” method [^8^](#_ENREF_8).. Multiple regressions and multiple correspondence analyses were performed in R environment (ver. 3.3.0, ref: R core team 2016) using FactoMineR [^9^](#_ENREF_9) and ggplot2 [^10^](#_ENREF_10) libraries.

**Supplementary Figure Legends**

**Suppl. Figure 1.** Analysis of A-1210477 in combination with ABTs on U937 cells and TF-1 cells. U937 and TF-1 cells were treated with 5 μM A-1210477 alone or in combination with 0.01 (**A-C**) or 0.1 (**B-D**) μM ABT-199 or ABT-263 for 18 h. Statistical analysis (three independent experiments) was performed with two-way ANOVA test (post-hoc test: Dunnett). Significance is reported as */^&^P < 0.05, **/^&&^P < 0.01, *** P< 0.001, ****/^&&&&^ P< 0.0001.

**Suppl. Figure 2.** Effect of combination of 18 h simultaneous treatment of 15 nM UNBS1450 and 0.01 µM ABT-199 on U937. Apoptosis was assessed by quantification of apoptotic cells by nuclear morphology after Hoechst staining and mitochondrial membrane potential loss assessed by MitoTracker® Red staining/FACS analysis. Statistical analysis (three independent experiments) was performed with two-way ANOVA test (post-hoc test: Dunnett). Significance is reported as ****/^&&&&^ P< 0.0001.

**Suppl. Figure 3.** Impairment of the replicative ability of U937 cells by UNBS1450/ABT-199 combination treatment. Colony formation capacity was strongly reduced when U937 cells were treated for at least 10 hours with a combination of UNBS1450 (30 nM) and ABT-199 (100 nM), Colonies were detected after 10 days of culture by adding 1 mg/ml of 3-(4,5-dimethylthiazol-2-yl)-2,5-diphenyltetrazoliumbromide (MTT) reagent (Sigma) and were scored by Image J software (U.S. National Institute of Health, Bethesda, MD, USA). Statistical analysis (three independent experiments) was performed with two-way ANOVA test (post-hoc test: Tukey). Significance is reported as ***/### P< 0.001.

**Suppl. Figure 4.** Analysis of the impact of the combination of ABT-199 or ABT-263 and UNBS1450 on healthy CD34^+^. Differential toxicity on CD34^+^ of the indicated combinations was assessed by analysis of cell proliferation by Trypan blue assay (**A, B**, top-left panels); quantification of fragmentation nuclei after Hoechst staining (**A, B**, middle-left panels), cells with loss of MMP (**A, B**, bottom-left panels), and positive to Annexin-V (**B**, bottom-right panels). Annexin-V assay was performed also on platelets at the concentrations indicated (24h of simultaneous treatment). Statistical analysis (three independent experiments) was performed with two-way ANOVA test (post-hoc test: Sidak; repeated measures). Significance is reported as * P< 0.05; * P< 0.05.

**Suppl. Figure 5**. Effects of ABT-199 on AML patient blasts. Cells were exposed as described in figure 2C. All correlations were tested by using GraphPad prism. Significance is reported as */P< 0.05; **/P< 0.01, ***/P< 0.001; ****/P< 0.0001 (two-way ANOVA; repeated measures; post-hoc analyses Dunnett; Sidak).

**Suppl. Figure 6**. Correlation between FLT3-ITD mutation and drug response. The impact of FLT3-ITD (ITD) *vs*. wild type (WT) on response to ABT-199, UNBS1450 and ABT-199+UNBS1450 was analyzed in *de novo* AML patients (Suppl. Table 1) by using GraphPad Prism (two-way ANOVA; post-hoc analyses Dunnett). Data corresponds to blasts in bulk, treated with concentrations of 10 nM ABT-199 and 30 nM UNBS1450 (single and combination analyses) after 48 h-pre-treatment UNBS1450.

**Suppl. Figure 7**. Association between response group and protein expression profiles. Groups were defined based on drug response: patients strongly responding to ABT-199 (group 1), patients strongly responding to UNBS1450 (group 2), patients responding to co-treatment (group 3), and patients not responding to any treatment (group 4). Protein expression levels were transformed to semi-quantitative scales (1: low <0.35, 2: middle, 3: high >0.7) and data were analyzed using multiple correspondence analysis (MCA). Interpretable points (with good representativity and contribution) were shown in dark blue.

**Suppl. Figure 8.** Differential expression of Mcl-1 proteins bands in established vs. primary cells. Comparison of Mcl-1 isoforms between the cell line U937 and selected primary samples exhibiting different Mcl-1 protein patterns. U937 cells were kept in normal culture conditions (referred as C.ed); alternatively they were thawed and processed for WB analysis following the same scheme adopted for primary samples (referred as T.ed).

**Supplementary references**

1. Juncker T, Cerella C, Teiten MH, Morceau F, Schumacher M, Ghelfi J*, et al.* UNBS1450, a steroid cardiac glycoside inducing apoptotic cell death in human leukemia cells. *Biochem Pharmacol* 2011 Jan 1; **81**(1)**:** 13-23.

2. Radogna F, Cerella C, Gaigneaux A, Christov C, Dicato M, Diederich M. Cell type-dependent ROS and mitophagy response leads to apoptosis or necroptosis in neuroblastoma. *Oncogene* 2015 Dec 7.

3. Florean C, Schnekenburger M, Lee JY, Kim KR, Mazumder A, Song S*, et al.* Discovery and characterization of Isofistularin-3, a marine brominated alkaloid, as a new DNA demethylating agent inducing cell cycle arrest and sensitization to TRAIL in cancer cells. *Oncotarget* 2016 Mar 19.

4. Grigorakaki C, Morceau F, Chateauvieux S, Dicato M, Diederich M. Tumor necrosis factor alpha-mediated inhibition of erythropoiesis involves GATA-1/GATA-2 balance impairment and PU.1 over-expression. *Biochem Pharmacol* 2011 Jul 15; **82**(2)**:** 156-166.

5. Souers AJ, Leverson JD, Boghaert ER, Ackler SL, Catron ND, Chen J*, et al.* ABT-199, a potent and selective BCL-2 inhibitor, achieves antitumor activity while sparing platelets. *Nat Med* 2013 Feb; **19**(2)**:** 202-208.

6. Vogler M, Hamali HA, Sun XM, Bampton ET, Dinsdale D, Snowden RT*, et al.* BCL2/BCL-X(L) inhibition induces apoptosis, disrupts cellular calcium homeostasis, and prevents platelet activation. *Blood* 2011 Jun 30; **117**(26)**:** 7145-7154.

7. Chou TC. Drug combination studies and their synergy quantification using the Chou-Talalay method. *Cancer Res* 2010 Jan 15; **70**(2)**:** 440-446.

8. Slinker BK. The statistics of synergism. *J Mol Cell Cardiol* 1998 Apr; **30**(4)**:** 723-731.

9. Lê S, Josse J, Husson F. FactoMineR: An R Package for Multivariate Analysis. *2008* 2008 2008-03-18; **25**(1)**:** 18.

10. Wickham H. *ggplot2: Elegant Graphics for Data Analysis*. Springer Publishing Company, Incorporated, 2009, 216pp.
