# Supplementary material for: Bcl-2 protein family expression pattern determines synergistic pro-apoptotic effects of BH3 mimetics with hemisynthetic cardiac glycoside UNBS1450 in acute myeloid leukemia
Source: Leukemia. 2017 Jan 3;31(3):755–9. doi: 10.1038/leu.2016.341 (PMC5339427; doi:10.1038/leu.2016.341)
Supplement: Supplementary Figures [file leu2016341x3.ppt]

## Slide 1
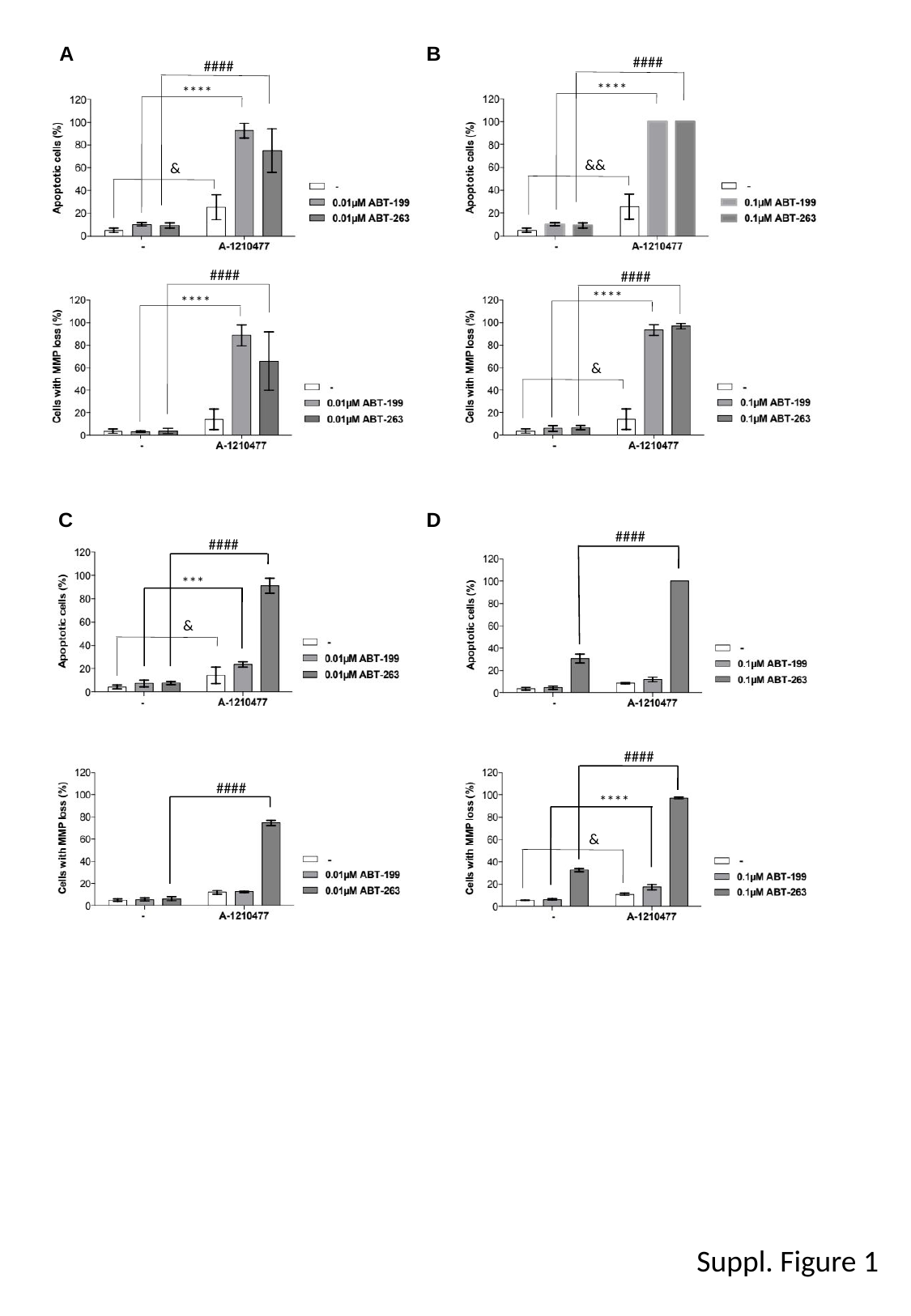

A
B
####
####
****
****
&&
&
####
####
****
****
&
C
D
####
####
***
&
####
****
####
&
Suppl. Figure 1

## Slide 2
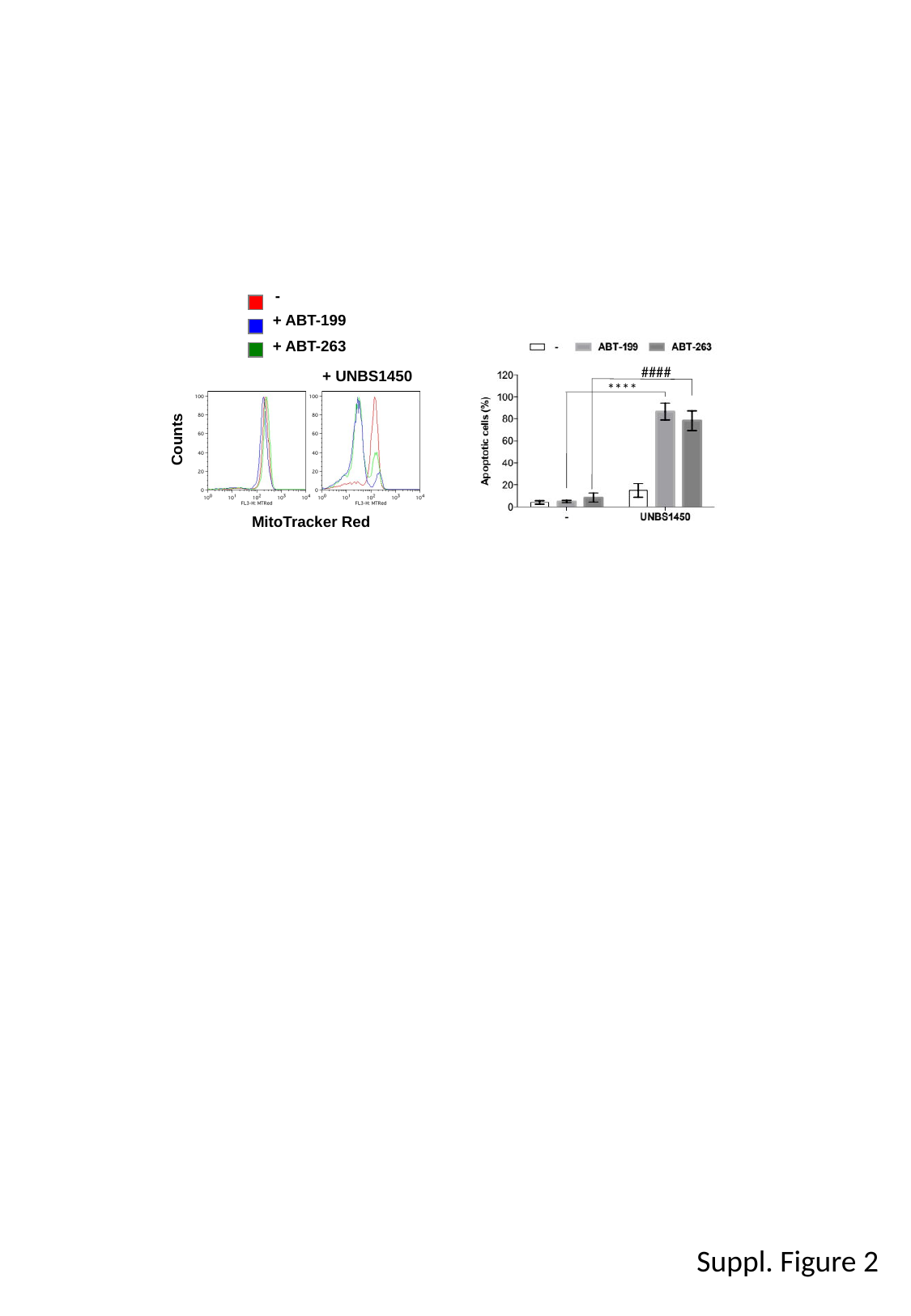

-
+ ABT-199
+ ABT-263
####
****
+ UNBS1450
Counts
MitoTracker Red
Suppl. Figure 2

## Slide 3
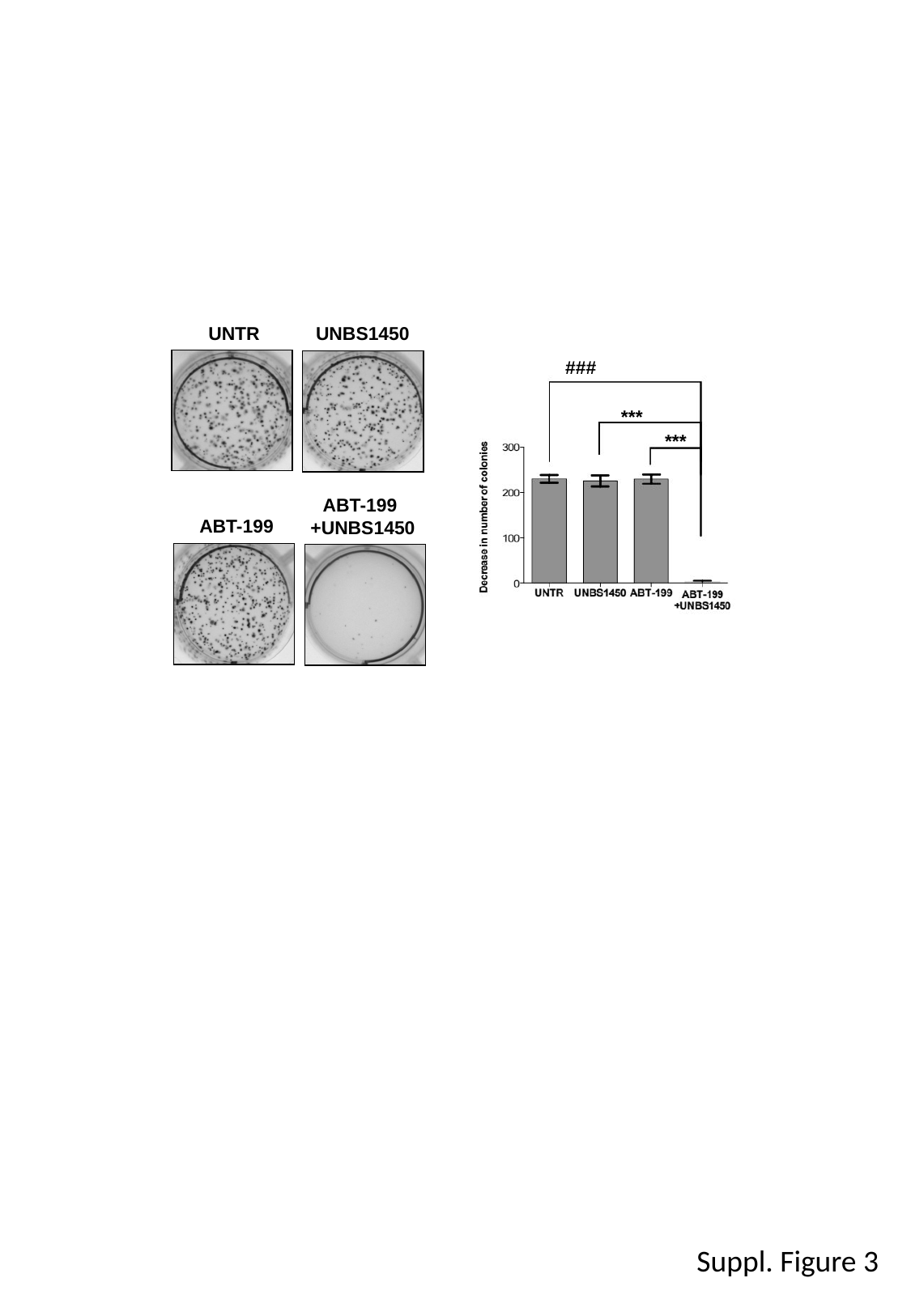

UNTR
UNBS1450
###
***
***
ABT-199
+UNBS1450
ABT-199
Suppl. Figure 3

## Slide 4
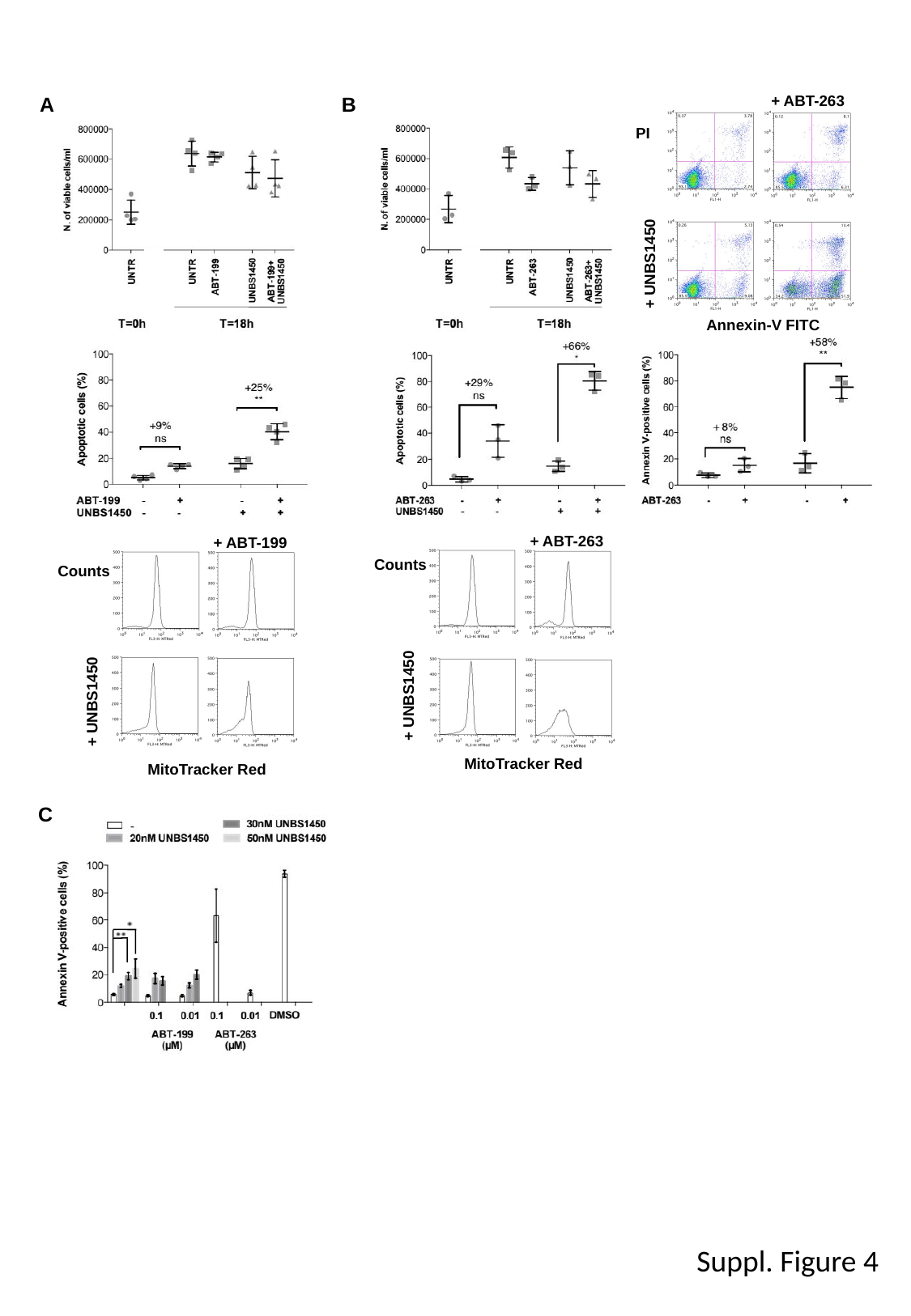

+ ABT-263
PI
+ UNBS1450
Annexin-V FITC
A
B
+ ABT-263
Counts
+ UNBS1450
MitoTracker Red
+ ABT-199
Counts
+ UNBS1450
MitoTracker Red
C
Suppl. Figure 4

## Slide 5
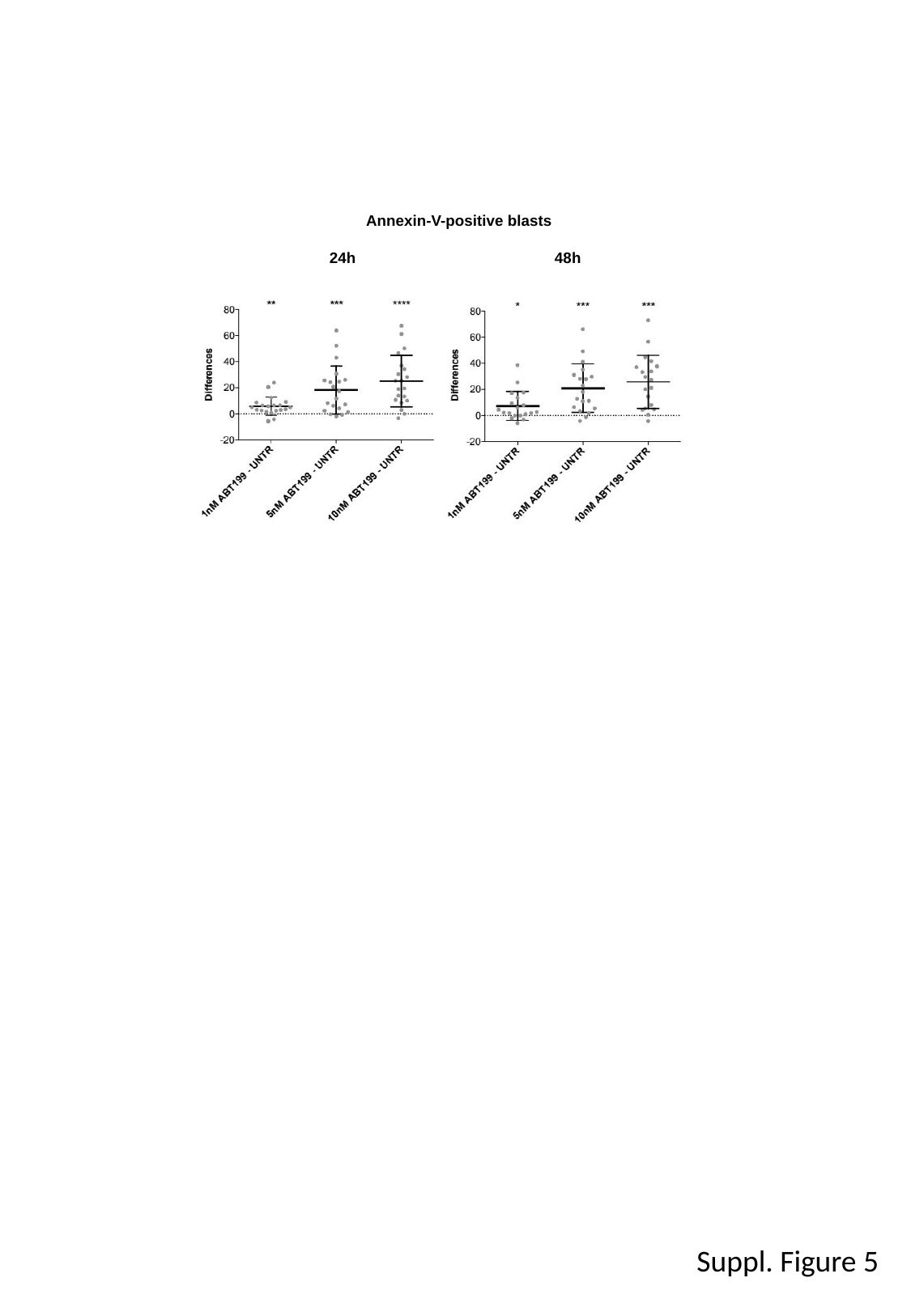

Annexin-V-positive blasts
24h
48h
Suppl. Figure 5

## Slide 6
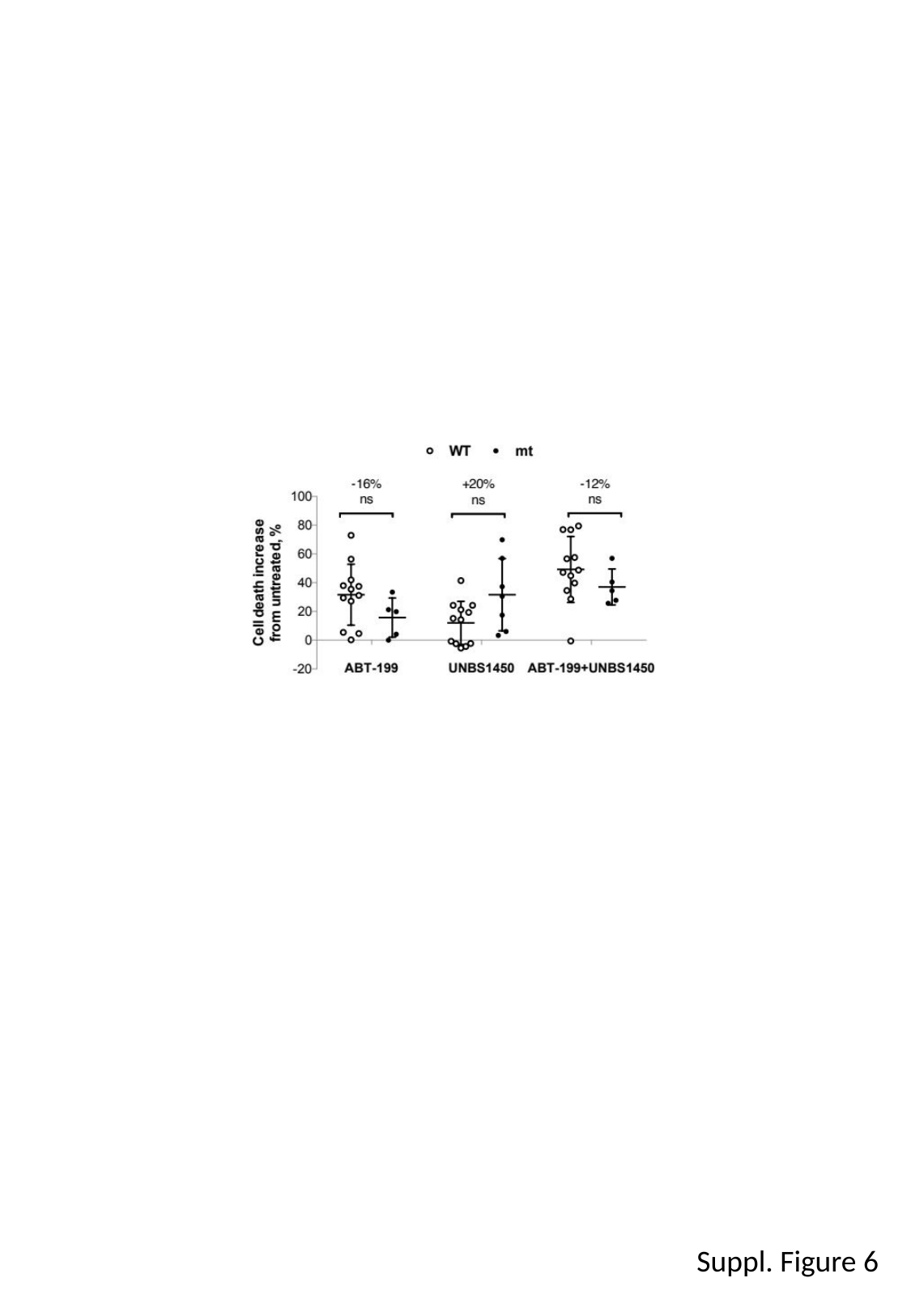

Suppl. Figure 6

## Slide 7
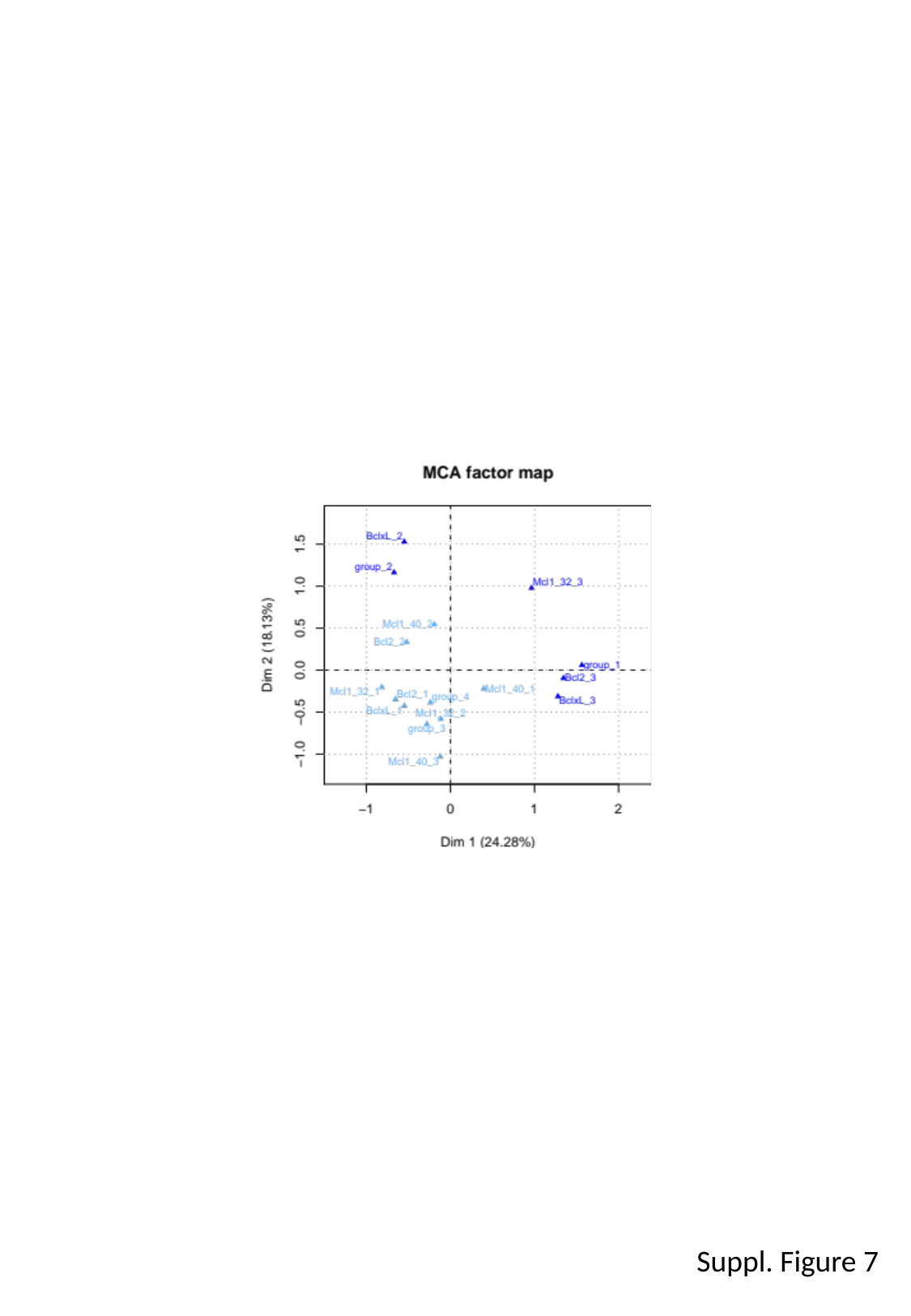

Suppl. Figure 7

## Slide 8
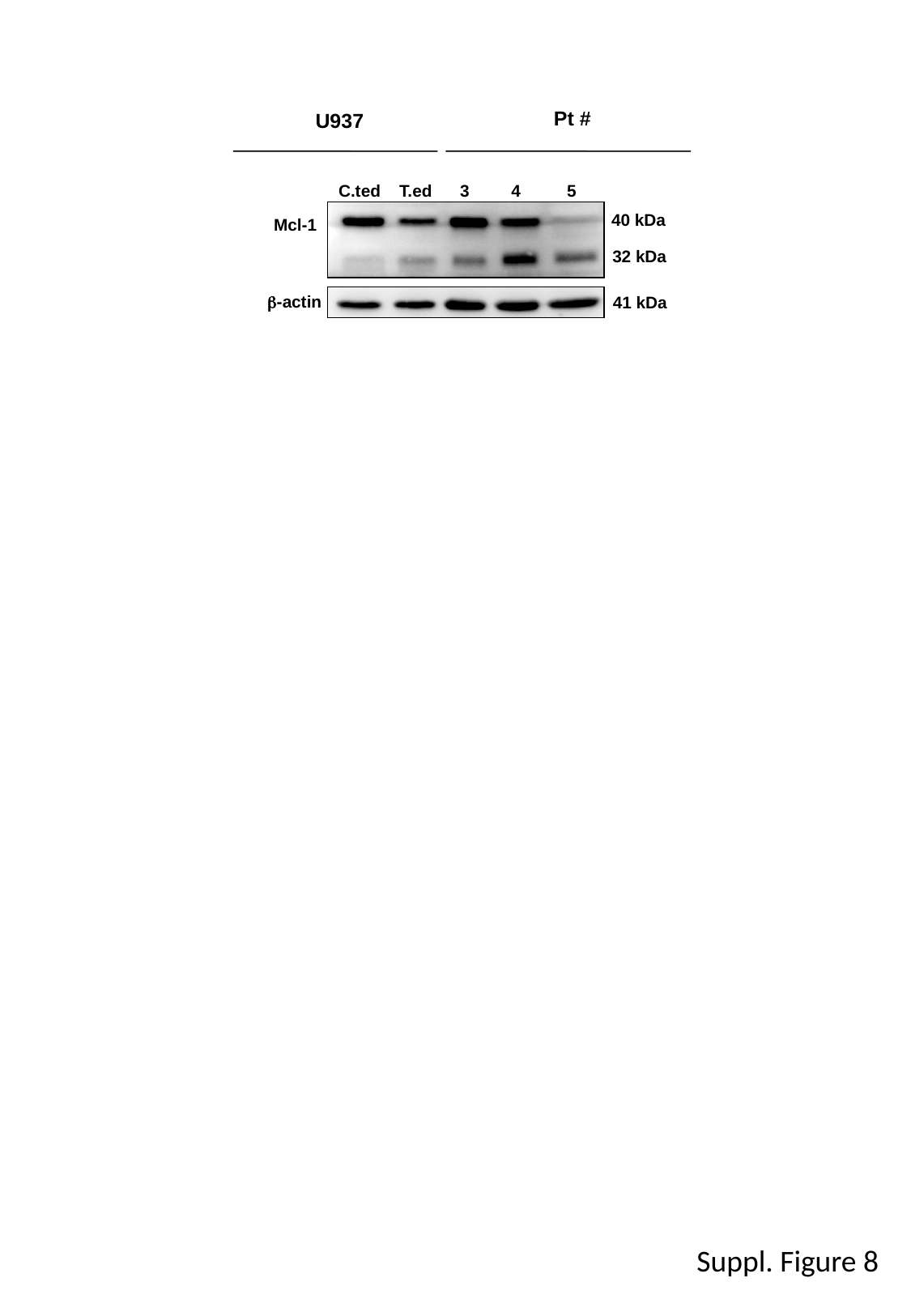

Pt #
U937
C.ted T.ed 3 4 5
40 kDa
Mcl-1
32 kDa
β-actin
41 kDa
Suppl. Figure 8
